# Case Report of Successful Extracorporeal CPR (eCPR) in Refractory Cardiac Arrest Caused by Fulminant Pulmonary Embolism with Remarkable Recovery

**DOI:** 10.3390/reports8030100

**Published:** 2025-06-25

**Authors:** Lukas Harbaum, Klevis Mihali, Felix Ausbüttel, Bernhard Schieffer, Julian Kreutz

**Affiliations:** Department of Cardiology, Angiology, and Intensive Care Medicine, University Hospital, Philipps Universität Marburg, 35043 Marburg, Germany

**Keywords:** out-of-hospital cardiac arrest (OHCA), extracorporeal cardiopulmonary resuscitation (eCPR), pulmonary embolism (PE)

## Abstract

**Background and Clinical Significance**: Fulminant pulmonary embolism (PE) leading to an out-of-hospital cardiac arrest (OHCA) is associated with a high mortality rate and cardiopulmonary resuscitation (CPR) frequently failing to achieve return of spontaneous circulation (ROSC). Extracorporeal CPR (eCPR) has emerged as a potential life-saving intervention. **Case Presentation**: A 66-year-old woman suffered an OHCA due to massive PE, presenting with pulseless electrical activity (PEA). After 90 min of pre- and in-hospital CPR without sustained ROSC, venoarterial extracorporeal membrane oxygenation (va-ECMO) was initiated as eCPR upon arrival at the hospital. Even after implantation of the va-ECMO, there was initially a pronounced acidosis (pH 6.9) with a high elevated lactate level (>30 mmol/L); these factors, together with the prolonged low-flow period, indicated a poor prognosis. Further diagnostic tests revealed intracranial hemorrhage (subdural hematoma), and systemic lysis was not possible. With persistent right heart failure, surgical thrombectomy was performed during hospitalization. Intensive multidisciplinary management finally led to successful therapy and weaning from mechanical ventilation, as well as to complete neurological recovery (CPC-Score 1-2). **Conclusions**: This case illustrates that eCPR can facilitate survival with good favorable neurological outcomes despite initially poor prognostic predictors. It underscores the importance of refining patient selection criteria and optimizing management strategies for eCPR in refractory cardiac arrest secondary to PE.

## 1. Introduction and Clinical Significance

Fulminant pulmonary embolism (PE) is a leading cause of cardiovascular death and a critical trigger of sudden cardiac arrest [1]. The acute obstruction of the pulmonary arteries—typically by a thrombus originating in the deep veins of the lower extremities—can rapidly lead to right ventricular failure, severe hypoxemia, and cardiac arrest [2]. Out-of-hospital cardiac arrest (OHCA) due to PE has an exceptionally high mortality rate because conventional cardiopulmonary resuscitation (CPR) often fails to generate sufficient circulation past the obstructive thrombus [3].

Extracorporeal cardiopulmonary resuscitation (eCPR) using venoarterial extracorporeal membrane oxygenation (va-ECMO) has emerged as a potential rescue strategy for patients with refractory cardiac arrest caused by reversible conditions, including massive PE [4]. While va-ECMO can provide temporary circulatory support and facilitate definitive interventions such as thrombectomy or catheter-directed thrombolysis, its efficacy remains highly dependent on early initiation and patient selection [5]. Prognostic factors such as initial cardiac rhythm, duration of low-flow, pH, and serum lactate levels play a crucial role in determining outcome [6]. Despite promising observational data, randomized controlled trials of eCPR remain scarce and standardized criteria for its use in OHCAs are still evolving. While patients with initial shockable rhythms and shorter low-flow times tend to have better outcomes [7], prolonged resuscitation with severe acidosis and hyperlactatemia is generally associated with poor survival [8]. However, in selected cases, intensive management, including early implementation of va-ECMO and advanced therapeutic strategies, may still lead to favorable neurological recovery. This case highlights the potential role of eCPR in refractory cardiac arrest due to massive PE and underlines the importance of timely multidisciplinary decision-making. It illustrates that va-ECMO may be a reasonable treatment option even in patients with poor initial prognostic indicators.

## 2. Case Presentation

A 66-year-old female patient with no significant medical history presented with a two-day history of progressive fatigue and intermittent dyspnea. Shortly before experiencing sudden cardiac arrest, the patient developed transient speech disturbances and circulatory weakness. The witnessed collapse led to immediate bystander-initiated CPR, which was continued by the Emergency Medical Services (EMSs) on arrival. In the patient’s region, the EMS control center provides real-time telephone guidance to bystanders in cardiac arrest situations to support early resuscitation.

The initial cardiac rhythm was pulseless electrical activity (PEA) and standard resuscitation protocols for non-shockable rhythms were implemented. Despite immediate initiation of CPR, the patient remained in PEA. The first return of spontaneous circulation (ROSC) occurred at 12 min after EMSs arriving but was not sustained. Intubation was performed with a video laryngoscope and mechanical CPR (mCPR) with corpuls cpr (GS Elektromedizinische Geräte G. Stemple GmbH, Kaufering, Germany) was initiated. After intubation, chest compression synchronized ventilation (CCSV) was performed (Weinmann Emergency Technology, Hamburg, Germany). Two transient episodes of ROSC were observed, but sustained hemodynamic stability was not achieved. After 55 min of pre-hospital resuscitation according to established guidelines, the patient was transferred under ongoing resuscitation measures for another 20 min to the emergency department of the University Hospital of Marburg. Upon arrival, the patient remained in refractory PEA. Due to the repeated phases of ROSC, the prompt initiation of resuscitation by a bystander, and the absence of relevant comorbidities, it was decided by consensus of the emergency team to perform eCPR. The va-ECMO (Maquet-Getinge GmbH, Rastatt, Germany) was implanted without complications via the right femoral artery (17 F sheath) and vein (21 F sheath), with antegrade leg perfusion ensured by the placement of an additional antegrade sheath. The va-ECMO was implanted in the cardiac catheterization laboratory under fluoroscopic guidance to ensure optimal cannula placement. The decision-making process had to occur within a very short timeframe due to prolonged resuscitation efforts and the resulting extreme time pressure. Considering the critical hemodynamic instability and refractory cardiac arrest, va-ECMO was considered the only remaining viable therapeutic option. Post-ROSC echocardiography showed severely reduced left ventricular function and the electrocardiogram (ECG) showed a new left bundle branch block (QRS 142 ms). In the absence of previous cardiac disease, immediate coronary angiography was performed, which ruled out coronary artery disease as the cause of the arrest. Epinephrine was subsequently tapered and arterial blood gas analysis after ROSC showed partial metabolic recovery after a period of approximately three hours (Table 1) with adequate oxygenation.

**Figure 1:**
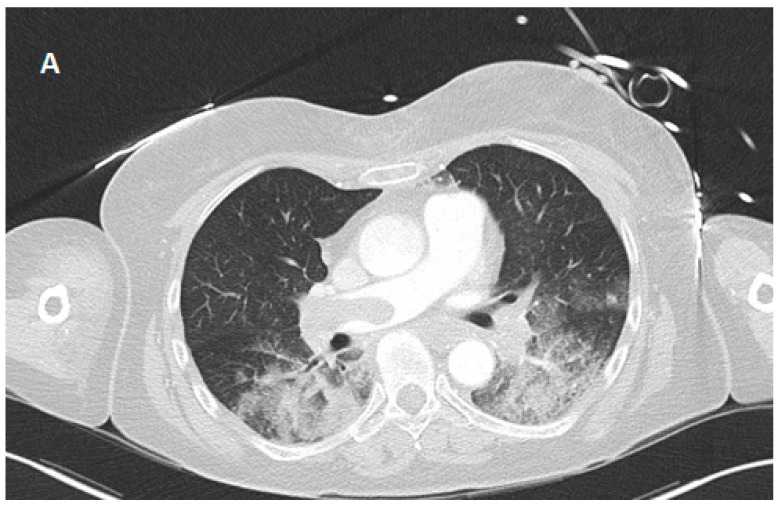

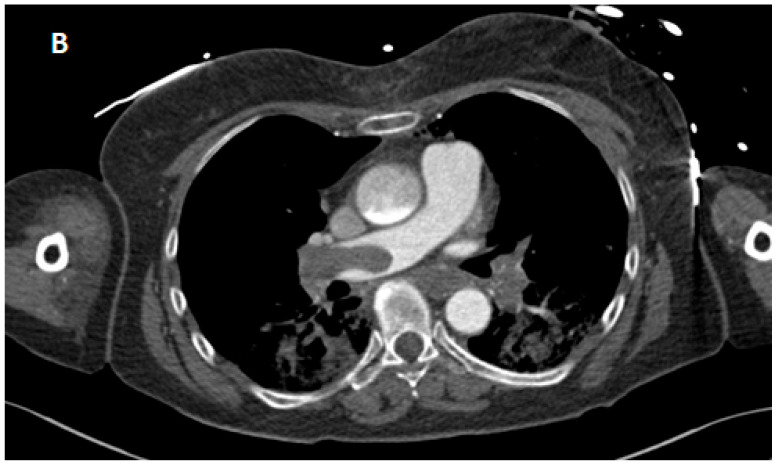
Chest computed tomography on the first day of hospitalization with evidence of central pulmonary artery embolism. (**A**) Lung window; (**B**) soft tissue window.

A whole-body computed tomography (CT) scan revealed massive bilateral PE as the most likely etiology (Figure 1). Additional findings included extensive lung consolidation (suggestive of aspiration/infarction pneumonia), serial rib fractures without pneumothorax, and a right prefrontal subdural hematoma (SDH). Due to the presence of an SDH, systemic thrombolysis was contraindicated in this patient.

Initial va-ECMO flow was set at 3.5–4.0 L/min with a target mean arterial pressure of >65 mmHg. Respiratory function and metabolic status were continuously assessed through serial measurements of arterial blood gases and lactate concentrations. Despite initial severe acidosis and high lactate, rapid improvement was observed within the first 24 h, suggesting effective hemodynamic support. The decision to initiate anticoagulation with unfractionated heparin was based on the ability to tightly control and adjust dosing through regular monitoring as is used as standard during va-ECMO treatment. Activated partial thromboplastin time (aPTT) in the range of 50–60 s was measured every 12 h to minimize both bleeding risk and the likelihood of thromboembolic complications. Importantly, serial cranial CT scans over the first 7 days showed no progression of the subdural hematoma. In addition, the presence of massive pulmonary embolism (PE) was considered, which represented a strong indication for anticoagulation and outweighed the risk of potential intracranial bleeding progression in this context.

**Figure 2:**
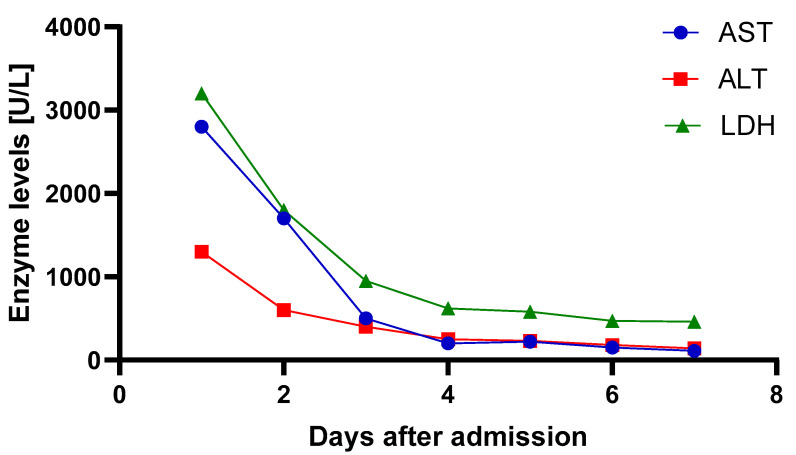
Transaminase and lactate dehydrogenase (LDH) values during the first week after va-ECMO implantation. Abbreviations: AST—aspartate aminotransferase; ALT—alanine aminotransferase; LDH—lactate dehydrogenase.

The metabolic status improved within 24 h, with lactate decreasing from 22 mmol/L to 2.2 mmol/L. Furthermore, there was a progressive, significant decline in the initially elevated transaminase and lactatdehydrogenase (LDH), as shown in Figure 2, which indicates improved tissue oxygen supply after ischaemic liver damage following CPR.

Targeted temperature management (TTM) was initiated by cooling the patient to 34 °C for 24 h using the Thermogard platform (TGXP, Zoll Medical, Cologne, Germany), followed by controlled rewarming at 0.25 °C/h to 37 °C. This target temperature was maintained for a further 24 h before the device was removed. A repeat echocardiographic examination after 48 h revealed a severely reduced left ventricular ejection fraction (35%) and right ventricular dysfunction, including paradoxical septal motion. Neuron-specific enolase (NSE) was measured several times in the first few days after admission and showed a peak value of 47 µg/L.

Given the persistent hemodynamic instability and lack of thrombus resolution on follow-up CT, a multidisciplinary team recommended surgical thrombectomy (Trendelenburg procedure). Postoperatively, the patient was hemodynamically stable with improved gas exchange. Anticoagulation with unfractionated heparin was continued with a target activated partial thromboplastin time (aPTT) of 50–60 s. Due to persistently elevated inflammatory markers despite initial broad-spectrum antimicrobial therapy (ampicillin/sulbactam, later escalated to piperacillin/tazobactam and levofloxacin), escalation to meropenem and vancomycin was required in accordance with clinical deterioration. In the case of acute renal failure with oliguria and significantly elevated renal retention parameters, continuous renal replacement therapy (CRRT) was initiated. In addition, hyperbilirubinemia was observed in the patient with a combined etiology of hemolysis during va-ECMO and initial massive right heart stress. Within five weeks, bilirubin had normalized to 2.38 mg/dL.

During the further inpatient course, echocardiographic monitoring showed a decreasing strain on the right heart. After 22 days, va-ECMO was successfully discontinued. The patient underwent a tracheostomy for prolonged ventilation and was gradually weaned from ventilatory support. On hospital day 37, the patient was awake, alert, neurologically responsive, and hemodynamically stable. During the ICU stay, a high-grade atrioventricular (AV) block (III°) was diagnosed, requiring pacemaker implantation. It occurred after catecholamine withdrawal and without previous bradyarrhythmias, suggesting a previously unrecognized conduction defect. The patient was transferred to a neurological rehabilitation center for further ventilatory weaning and functional recovery. Despite an initially poor prognosis (Pulmonary Embolism Severity Index (PESI) score: 216; estimated 30-day mortality: 10–24.5%), the patient achieved a favorable outcome with preserved neurological function (CPC-Score 1-2, mRS-Score 2). The patient was mobilized with the help of physiotherapy and demonstrated the ability to respond appropriately to simple yes/no questions. Additionally, she had started performing basic personal hygiene tasks independently. Based on these clinical observations from the last two days in our hospital, we assessed the neurological outcome as lying between CPC 1 and CPC 2, reflecting good cerebral performance with minor cognitive or functional limitations.

**Figure 3:**
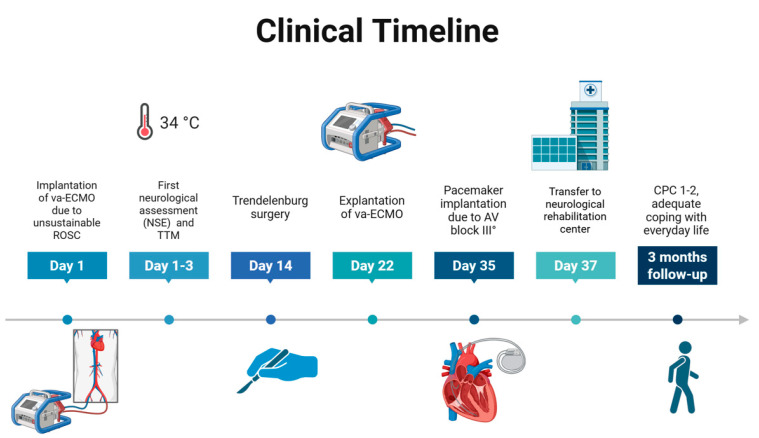
**Clinical timeline.** A timeline of events from hospital admission with immediate implantation of va-ECMO through surgery to final discharge to neurological rehabilitation center. Abbreviations: va-ECMO—venoarterial extracorporeal membrane oxygenation; ROSC—return of spontaneous circulation; NSE—neuron-specific enolase; TTM—targeted temperature management; AV-block—atrioventricular block; CPC—cerebral performance category.

**Figure 4:**
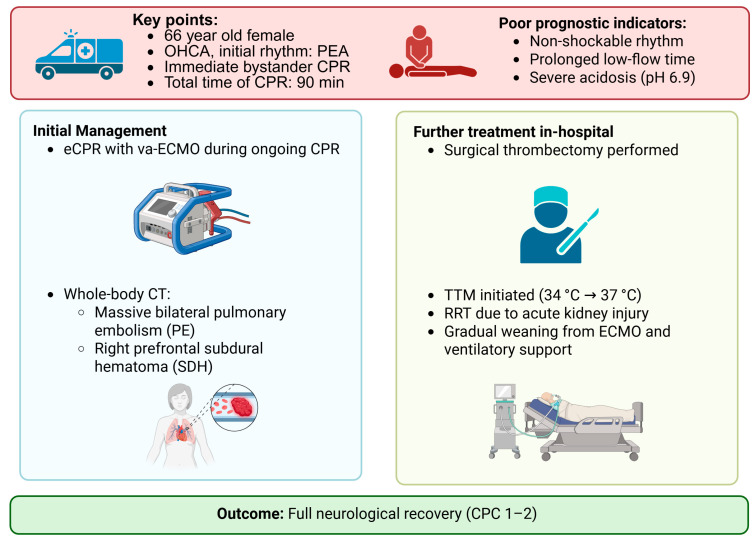
**Clinical course and key interventions.** Review of the clinical course of a 66-year-old woman with out-of-hospital cardiac arrest (OHCA) due to pulmonary embolism (PE). After 90 min of CPR and initiation of eCPR with va-ECMO, CT revealed bilateral PE and a subdural hematoma (SDH). Surgical thrombectomy, targeted temperature management (TTM), and renal replacement therapy (RRT) were performed. Despite poor prognostic factors, the patient achieved full neurological recovery (CPC 1-2). Abbreviations: OHCA—out-of-hospital cardiac arrest; PE—pulmonary embolism; CPR—cardiopulmonary resuscitation; eCPR—extracorporeal cardiopulmonary resuscitation; va-ECMO—venoarterial extracorporeal membrane oxygenation; CT—computed tomography; SDH—subdural hematoma; TTM—targeted temperature management; RRT—renal replacement therapy; CPC—cerebral performance category.

Accordingly, a modified Rankin Scale (mRS) score of 2 (slight disability) was also assigned to describe her functional independence with some remaining limitations. At the three-month follow-up, the patient remained alert and fully oriented, with preserved cognitive function and ongoing neurological recovery. While she still required some assistance with activities of daily living, she continued to make functional progress and showed no evidence of significant neuropsychological impairment. The clinical course and key interventions are summarized in Figure 3 and Figure 4.

## 3. Discussion

This case suggests that early initiation of bystander resuscitation and high-quality EMS care may be important factors in OHCA survival. Good pre-clinical technical equipment with video laryngoscopy, which allows fast and reliable intubation, systems for mCPR during transport, and automated ventilation modes developed for CPR, such as CCSV, can also play a crucial role. Meta-analyses show that the use of a video laryngoscope results in a significantly higher rate of successful intubations on the first attempt, especially for less-experienced emergency physicians, and that secondary complications can be prevented [9,10,11]. Furthermore, CCSV has shown potential advantages over conventional modes during prolonged CPR, particularly in improving cerebral oxygenation and perfusion [12]. Implantation of va-ECMO systems during ongoing resuscitation requires well-trained teams and standardized protocols in cardiac arrest centers (CACs) for the post-resuscitation phase.

Extracorporeal cardiopulmonary resuscitation (eCPR) is an emerging rescue therapy for refractory cardiac arrest due to reversible causes such as massive PE [13]. Patient selection is critical, with favorable outcomes associated with a shockable rhythm, short low-flow time [14], and signs of life during CPR [6]. However, this case presented several poor prognostic factors, including prolonged resuscitation (90 min), non-shockable rhythm, severe acidosis (pH 6.9), and extreme hyperlactatemia (>30 mmol/L), all of which are typically associated with low survival rates [5]. Despite multiple unfavorable prognostic indicators, the initiation of eCPR was deemed appropriate due to the prompt initiation of basic life support and the presence of a potentially reversible cause [15].

A comparison with existing reports of eCPR in massive PE reveals significant variability in patient selection, timing, and outcomes. Meta-analyses and registry data [6,16] suggest that overall, survival after eCPR in PE-related cardiac arrest remains low, but favorable outcomes are possible, particularly with early intervention and a reversible pathology. Current guidelines, such as those by the European Resuscitation Council (ERC) [17], support eCPR in cases where the cause is potentially reversible, including massive PE. However, standardized protocols are still being developed. Our case differs from the typical inclusion criteria, such as a shockable rhythm and a short low-flow time, yet it confirms the findings of Stadlbauer et al. [18], who reported improved outcomes when reperfusion therapies, such as surgical thrombectomy, were applied alongside va-ECMO. Our case supports this as surgical thrombectomy after eCPR resulted in full recovery despite contraindication to thrombolysis.

This case highlights the importance of individualized decision-making, especially in specialized CACs. Emerging pre-hospital strategies, such as the “load-and-go” approach, emphasize rapid transport to eCPR-capable hospitals rather than prolonged resuscitation in the field [13]. A meta-analysis has shown a survival benefit of eCPR in patients with cardiac arrest due to PE, regardless of the subsequent management strategy [19].

In addition, the use of subsequent TTM may have contributed to the favorable neurological outcome, as suggested by data showing improved outcomes in patients with OHCA and eCPR [20]. In this case, va-ECMO provided hemodynamic stabilization, but unresolved right heart failure necessitated surgical thrombectomy. Systemic thrombolysis was contraindicated due to the presence of a subdural hematoma, highlighting the complexity of anticoagulation management in critically ill patients. In this context, early whole-body CT imaging—including cranial scans—performed immediately after ROSC was essential for timely diagnosis and therapeutic decision-making. This highlights the importance of early imaging in eCPR patients to balance bleeding risks with appropriate reperfusion strategies. Despite severe metabolic derangements, the patient achieved a favorable neurological outcome, highlighting the potential of eCPR in high-risk scenarios [21]. This case did not meet standard eCPR eligibility criteria and illustrates the therapeutic complexity arising from the coexistence of massive pulmonary embolism and intracranial hemorrhage. This underlines the necessity of individualized clinical decision-making in selected cases considered for eCPR.

## 4. Conclusions

In this case, timely ECMO support, multidisciplinary care, and advanced interventions may have contributed to survival with preserved neurological function. Further research is needed to refine patient selection and optimize pre-hospital strategies for eCPR in PE-induced cardiac arrest.

## 5. Limitations

This report describes a single patient who achieved favorable neurological recovery despite multiple poor prognostic factors. Although this result is encouraging, it cannot be generalized. Clear criteria for the use of eCPR are essential, but individual factors such as timing, comorbidities, and reversibility of the underlying cause must always be considered in clinical decision-making.

## Figures and Tables

**Table 1:** Blood gas analyses of the patient at the following times: 1. immediately after va-ECMO implantation; 2. 3 h after va-ECMO implantation; 3. 24 h after va-ECMO implantation. Arterial blood gas was measured from the left femoral artery at time points 1 and 2 and from the right radial artery at time point 3. Abbreviations: ABG—arterial blood gas; BE—base excess; pO_2_—partial pressure of oxygen; pCO_2_—partial pressure of carbon dioxide.

	ABG 1 (Immediately After Va-ECMO Implantation)	ABG 2 (3 h After Va-ECMO Implantation)	ABG 3 (24 h After Va-ECMO Implantation)
pH	6.90	7.30	7.52
pCO_2_ (mmHg)	34.5	17.9	29.0
pO_2_ (mmHg)	423	312	121
Lactate (mmol/L)	>30.0	22.0	2.6
BE	−19.0	−16.4	+1.8

## Data Availability

The original contributions presented in this study are included in the article. Further inquiries can be directed to the corresponding author.
